# Monocyte Recruitment, Specification, and Function in Atherosclerosis

**DOI:** 10.3390/cells10010015

**Published:** 2020-12-24

**Authors:** Ki-Wook Kim, Stoyan Ivanov, Jesse W. Williams

**Affiliations:** 1 Department of Pharmacology and Regenerative Medicine, University of Illinois College of Medicine, Chicago, IL 60612, USA; Kiwook@UIC.edu; 2 INSERM U1065, Centre Méditerranéen de Médecine Moléculaire C3M, Université Côte Azur, 06204 Nice, France; Stoyan.IVANOV@unice.fr; 3 Center for Immunology, Department of Integrative Biology & Physiology, University of Minnesota Medical School, Minneapolis, MN 55455, USA

**Keywords:** atherosclerosis, monocyte, macrophage, inflammation, trafficking, cardiovascular disease

## Abstract

Atherosclerotic lesions progress through the continued recruitment of circulating blood monocytes that differentiate into macrophages within plaque. Lesion-associated macrophages are the primary immune cells present in plaque, where they take up cholesterol and store lipids in the form of small droplets resulting in a unique morphology termed foam cell. Recent scientific advances have used single-cell gene expression profiling, live-cell imaging, and fate mapping approaches to describe macrophage and monocyte contributions to pro- or anti-inflammatory mechanisms, in addition to functions of motility and proliferation within lesions. Yet, many questions regarding tissue-specific regulation of monocyte-to-macrophage differentiation and the contribution of recruited monocytes at stages of atherosclerotic disease progression remain unknown. In this review, we highlight recent advances regarding the role of monocyte and macrophage dynamics in atherosclerotic disease and identify gaps in knowledge that we hope will allow for advancing therapeutic treatment or prevention strategies for cardiovascular disease.

## 1. Introduction

Atherosclerosis presents in the mid- and large-sized arteries as a cholesterol-laden cellular aggregate, referred to as plaque [1]. Advanced stages of atherosclerosis lead to the formation of a vulnerable plaque with a necrotic core which, when it ruptures, contributes to many cardiovascular disease outcomes, including myocardial infarction and stroke. Collectively, cardiovascular disease remains a leading cause of morbidity and mortality worldwide, and improvements in prevention or treatment strategies for atherosclerosis will reduce these adverse outcomes [2]. Atherosclerosis is well known to be driven by the accumulation of cholesterol in the arterial intima, but it is also mediated in part through a chronic inflammatory response, constituted by a diverse range of immune cell infiltration [3,4]. This diversity includes cells from the innate and adaptive immune arms. However, the most common immune cells in expanding atherosclerotic plaque are monocytes and macrophages. These cells play prominent roles in cholesterol accumulation, lesion matrix remodeling, cytokine production, and clearance of dead cell debris. Thus, targeting plaque macrophages and their monocyte precursors is a potentially relevant therapeutic approach and of great interest to the atherosclerosis field [5]. In this review, we will focus on the development and recruitment of monocytes and their differentiation to plaque-associated macrophages. We will discuss recent findings and gaps in current knowledge that represent important opportunities for future research applications. 

## 2. Monocyte Development

### 2.1. Monocyte Subsets

Monocytes are continuously generated from hematopoietic stem cells (HSCs) in the bone marrow starting during embryogenesis and continuing throughout the lifespan in humans and mice. The heterogeneity of human monocytes was first reported in the late 1980s: CD14^+^ and CD14^lo^ CD16^+^ monocytes [6]. Then, CD14^+^ monocytes were further divided into CD14^+^ CD16^−^ and CD14^+^ CD16^+^ [7]. Murine monocytes can be identified through expression of M-CSFR (*Csf1r*/CD115) and CD11b *(Itgam)*. The generation of CX3CR1^gfp^ knock-in mice provided a breakthrough in defining the heterogeneity of murine monocyte subsets in bone marrow, blood, and other peripheral tissues [8,9]. In these mice, murine monocytes can be divided into two groups: CX3CR1^int^, Ly6C^hi^, CCR2^+^ CD62L^+^ CD43^int^ (called Ly6C^hi^ classical monocytes) and CX3CR1^hi^ Ly6C^lo^, CCR2^−^ CD62L^−^ CD43^hi^ (called Ly6C^lo^ nonclassical monocytes) [10]. Side-by-side comparison studies including transcriptomic analysis and surface marker expression revealed that murine Ly6C^hi^ monocytes and Ly6C^lo^ monocytes correspond to the CD14^+^ classical monocytes and CD14^lo^ CD16^+^ nonclassical monocytes in humans, respectively [11]. 

### 2.2. Developmental Pathway of Monocytes

**Figure 1:**
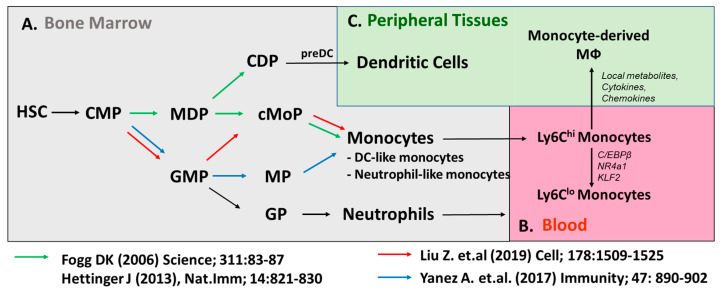
Developmental heterogeneity of monocyte populations. (**A**) Classical model (green arrow) of monocyte development shows monocytes are generated through Macrophage (Mφ) and DC Precursors (MDPs). Emerging evidence (red, blue arrows) suggest monocytes can be generated through the Granulocyte and Macrophage Progenitors (GMPs). Ly6C^hi^ monocytes give rise to Ly6C^lo^ monocytes in (**B**) blood or monocyte-derived macrophages in (**C**) peripheral tissues.

In the bone marrow, HSCs specify into common lymphoid progenitors (CLPs) for the differentiation of lymphocytes such as T, B, and NK cells and common myeloid progenitors (CMPs) for the differentiation of innate myeloid cells. Then, CMPs are able to give rise to the granulocyte and macrophage progenitors (GMPs: Lineage^−^, CD117(c-kit)^+^, CD135(Flt3)^−^, CD115(Csf1R)^lo^ FcγR^hi^). The major advancement for monocyte lineage study was the identification of macrophage/dendritic cell progenitors (MDPs: Lineage^−^, CD117(c-kit)^+^, CD135(Flt3)^+^, CD115(Csf1R)^+^,) which are likely derived from GMPs, as the further specification of MDPs is committed to the mononuclear phagocytes, not granulocytes [12]. Then, MDPs give rise to the common monocyte progenitor (cMoP), which is committed to the monocyte populations without differentiating to conventional dendritic cells (cDCs). Transition from cMOP to a bone-marrow-residing monocyte requires the expression of CXCR4 [13]. However, advanced techniques and newly generated transgenic animals have challenged this sequential pathway of monocyte development, as is outlined by the schematic in Figure 1. Using adoptive GMP and MDP transfer approaches, the Goodridge group proposed that MDPs are not strictly derived from GMPs, which have the potential to generate monocytes through the monocyte-committed progenitors (MPs). GMP-derived monocytes and MDP-derived monocytes are differently sensitive to microbial stimuli, implying monocytes have their own roles that are dependent on their origins [14,15]. Why these complementary pathways coexist and their relative contribution to monocyte pool dynamics in health and disease remain to be addressed.

Taking advantage of transgenic mice driven by the MS4a3 promoter specifically expressed in GMPs and their progeny, the fate mapping approach supports that MDPs are not the sole precursors to generate monocytes [16]. Alternatively, GMP-derived precursors were shown to generate monocytes directly without a transition into cMoP [14]. The concept of dual developmental origins of monocytes is strongly supported by an elegant in vitro and in vivo study with a combination of lineage tracing with single-cell RNA-Seq, which showed GMP-derived monocyte highly expressed neutrophil-related genes (called neutrophil-like monocytes), while MDP-derived monocytes were highly upregulated in DC-related genes (called DC-like monocytes) [17]. However, the differential/overlapping roles of these monocytes with distinct origins may have differential contributions to macrophage populations in atherosclerosis, and depending on disease state, will need to be elucidated in future studies.

### 2.3. Monocyte Maturation in Blood

Circulating blood monocyte numbers are closely associated with the formation and expansion of atherosclerosis in humans and preclinical mouse models [18,19,20]. This expansion in monocyte numbers is often referred to as monocytosis. A variety of factors, including stress, sleep, diet, environmental temperature, and caloric intake, have all been shown to control monocyte development [21,22,23,24,25]. Generated in bone marrow, CCR2-expressing Ly6C^hi^ classical monocytes emigrate to the blood vasculature by the chemotactic activity of CCL2 expressed in mesenchymal stem cells (MSCs) of bone marrow. In addition to marrow, during hypercholesterolemia, monocyte maturation has also been observed to occur in the spleen, a process known as extramedullary hematopoiesis [26]. Persisting only transiently in the blood with a short lifespan (0.8 days in mice, 1.6 days in humans), Ly6C^hi^ monocytes have the potential to extravasate to peripheral tissues or convert to Ly6C^lo^ monocytes in blood (Figure 1). Fate mapping approaches, adoptive monocyte transfer, and BrdU pulse-chase studies have shown that Ly6C^hi^ monocytes give rise to Ly6C^lo^ monocytes with an extended lifespan (<2.2 days) in blood and bone marrow [27]. Similar to murine monocytes, human classical monocytes sequentially differentiate to the nonclassical monocytes with a lifespan of 7 days through CD14^+^ CD16^+^ intermediate monocyte populations [28]. However, these studies have primarily been performed in healthy subjects, and whether cardiovascular disease influences the kinetics of monocyte maturation remains to be addressed. In addition, aging-associated changes in monocyte epigenetic status have been associated with gene expression changes in healthy humans, so future studies will need to address the conversion of these hypo- or hypermethylated loci in association with high-risk cardiovascular patients [29]. 

Ly6C^lo^ monocytes have distinct gene expression profiles and epigenetic profiles from Ly6C^hi^ monocytes. During the transition to Ly6C^lo^ monocytes, *C/EBPβ*, *Nr4a1*, and *Klf2* were found to be highly upregulated compared with Ly6C^hi^ monocytes [30,31]. Ly6C^lo^ monocytes were ablated in NR4a1-deficient mice and it turned out that the enhancer region (E2) of *Nr4a1* locus controls the development/maintenance of Ly6C^lo^ monocytes [30,32,33]. Deletion of the super enhancer region of the Nr4a1 gene also replicated ablation of nonclassical monocyte development, without the off-target effects in controlling lymphocytes seen in the total Nr4a1-deficient mice [30]. Mildner et al. demonstrated that C/EBPβ binds to the E2 enhancer region, which induces the expression of the *Nr4a1* gene [31]. Thus, Ly6C^lo^ monocytes were not generated in C/EBPβ-deficient mice, similar to NR4a1-deficient mice. Another study showed that the interaction of endothelial-derived Delta-like 1 (DII1) with Notch2 expressed on Ly6C^hi^ monocytes induced the transition of Ly6C^hi^ monocytes to Ly6C^lo^ monocytes [34]. 

## 3. Monocyte Recruitment and Specification

### 3.1. Nonclassical Monocyte Function in the Vasculature

Nonclassical monocytes play a key function in maintaining the vasculature through interactions and patrolling behavior with the arterial endothelium [33,35]. During atherosclerosis, CX3CR1, a receptor preferentially expressed on nonclassical monocytes, interacts with its sole ligand CX3CL1 (fracktalkine) to favor cell survival [36]. Additionally, this axis may play a role in localization within plaque [10]. In homeostatic conditions, Ly6C^lo^ monocytes have reduced potential to differentiate into tissue macrophages, with a few exceptions (including the interstitial lung macrophage subset [37]). They persist in vasculature by patrolling on the blood endothelial cells with crawling behavior dependent on LFA-1, CX3CR1, and CD36 [33,38,39]. Visualizing monocyte patrolling the endothelial surface through the use of intravital multiphoton microscopy, behavior has been a major advancement in the field [38,40]. Animals deficient in nonclassical monocytes, such as NR4A1 knockout mice [32], support the concept that nonclassical monocytes support endothelial health and maintenance [41,42]. In addition, Kindlin-3 was recently suggested to control the ability of nonclassical monocytes to interact with endothelium, while it was not required for survival, and to replicate many of these protective roles for nonclassical monocytes in promoting endothelial barrier homeostasis [43]. Thus, this model will allow for distinguishing the specific function of nonclassical monocyte–endothelial interactions, independent of other potential roles that nonclassical monocytes may play in circulation. 

### 3.2. Monocyte Recruitment to Atherosclerotic Plaque

In the steady state, classical monocytes are associated with recruitment into tissues and play an important role in maintaining tissue macrophage populations. Nonclassical monocytes are less efficiently recruited to lesions, which is known to be dependent in part on CCR5 expression [10]. While many tissue-resident macrophage populations reside independently of circulating progenitor cells during homeostasis, such as microglia, many other macrophage populations, such as MHCII^+^ peritoneal macrophages, are continuously replenished by circulating Ly6C^hi^ monocytes in homeostatic conditions [44,45]. Yet, in other tissues, macrophages originate in the embryo but require monocyte recruitment to fill the macrophage niche following birth, such as the aortic adventitia [46,47], or require a slow replacement from monocytes through the lifespan of the mouse, such as macrophages in the heart [48] and lungs [49] being replaced by monocyte-derived macrophages with age. Inflammatory conditions accelerate the generation of bone marrow monocytes and extravasation of blood monocytes to the inflammatory sites. Mobilization into inflamed tissues, such as an atherosclerotic plaque, is mediated by the local production of chemokines that bind to receptors on the monocyte to promote migration from the blood into the tissues. CCR2, CCR5, and CX3CR1 have all been shown to influence monocyte recruitment to atherosclerotic plaque and control disease outcome [18]. The best described is through the CCL2 (MCP-1): CCR2 axis, whereby CCL2 is highly expressed in plaque, driving classical monocyte recruitment into tissues. Other chemokine pairs have been described, including CCR5:CCL2/5, CX3CR1:CX3CL1, and CXCR4/CXCL12, which promote the development and recruitment of monocytes. Thus, control of the aforementioned chemokine axis may be an exciting therapeutic target to prevent monocyte recruitment to plaque for disease prevention.

Transendothelial migration is the arrest and migration across the endothelial layers [50]. This is an active process, requiring molecular signaling between endothelial cells on the artery lumen and the circulating monocytes. Monocytes express a variety of adhesion molecules such as LFA1, PSGL1, CD31, VLA-4, and CD62L which allow for adherence and transmigration to occur through interactions with their endothelial partners. Blocking approaches directed against CD11b, ICAM1, LFA-1, or VLA-4 have all shown dramatic defects in monocyte recruitment in models of atherosclerosis. Once monocytes access the peripheral tissues, they differentiate to macrophage populations and require CSF-1 for their survival and maintenance [51,52]. It was recently found that production of CSF-1 that supports plaque macrophage survival comes from local smooth muscle and endothelial cells, not from systemic production [53]. The topics of transition of Ly6C^hi^ monocytes to macrophages in pathological conditions (e.g., atherosclerosis) are discussed below.

## 4. Macrophage Diversity and Function in Atherosclerosis

### 4.1. Monocyte-to-Macrophage Differentiation and Role in Atherosclerosis

Monocyte extravasation into expanding atherosclerotic plaque requires their rapid adaptation to a new local microenvironment, sometimes referred to as a niche. This differentiation step requires signaling between aortic stromal cells and macrophages, including through the CSF-1 receptor (*Csf1r*, CD115) to maintain macrophage survival [51,52]. However, only limited studies have addressed the tissue-specific adaptation of monocytes within atherosclerotic plaque. Based on studies of monocyte function in other tissues and in plaque macrophages, monocyte differentiation and their ability to give rise to proinflammatory or anti-inflammatory macrophages depend on local metabolic cues and cytokine availability. For instance, glucose and lipids have been shown to modulate monocyte generation and macrophage functions during atherosclerosis establishment and progression [54,55,56,57,58,59]. Lesion-associated macrophages accumulate lipids through cholesterol uptake by scavenger receptors that are highly expressed on their surface and were described in early pioneering studies [60]. Key receptors involved in this uptake include SR-A1 and CD36, and this leads to the generation of “foam cells”, a characteristic macrophage morphology that is found at all stages of disease progression. In addition, lipid accumulation in nonmacrophage cells has also been identified and contributes to a substantial number of the total lipid-loaded cells in plaque [61,62,63]. The role and development of these cells are discussed elsewhere [64,65]. 

**Figure 2:**
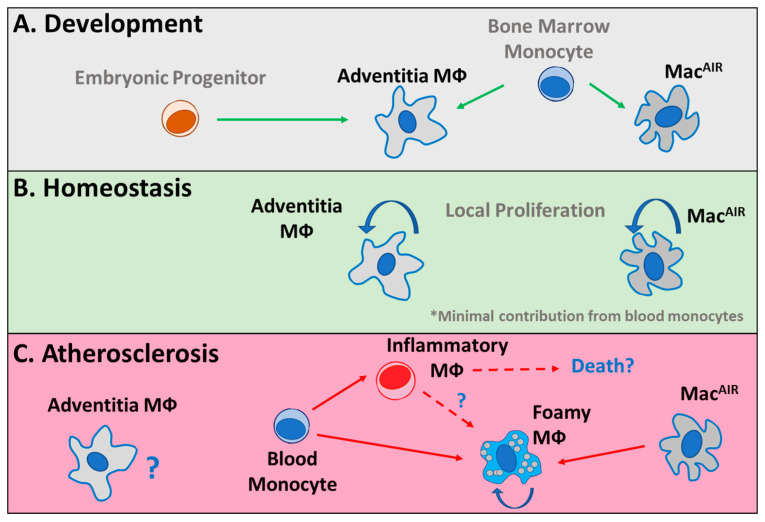
Monocyte and macrophage dynamics in the aorta. (**A**) Adventitia macrophages (Mφ) that develop during embryonic development and immediately following birth are complemented by a wave of monocytes. In the intima, aorta-intima-resident macrophages (Mac^AIR^) develop from bone marrow monocytes immediately following birth. (**B**) During homeostasis, adventitia and Mac^AIR^ are maintained in their respective tissues, independent of additional monocyte supplementation, through local proliferation. (**C**) During atherosclerosis, the role of adventitia macrophages is poorly described and their relationship with plaque-associated macrophages is unknown. During disease progression, Mac^AIR^ are the initial cells to differentiate into foamy macrophages. Subsequently, monocytes infiltrate into tissue and differentiate into inflammatory or foamy macrophages. The interactions between these monocyte-derived cell types and their potential contributions to cell death pathways are unclear.

In mouse models, adventitia-resident macrophages develop in the embryo and are supplemented by an influx of monocytes shortly after birth [46,47]. These cells are maintained in the tissue by local proliferation through the lifespan of the mouse. Furthermore, aorta-intima-resident macrophages (Mac^AIR^) reside in the aorta and derive from monocytes after birth [66,67,68]. Mac^AIR^ are the first population of foamy macrophages that can be detected within a week of high fat/high cholesterol feeding [66,69]. Following high fat/high cholesterol feeding, these cells also promote the recruitment of monocytes to plaque [66]. Interestingly, it was suggested that foamy macrophages derived from Mac^AIR^ or circulating monocyte origins converge on an overlapping gene expression program, indicating dual origins of a single macrophage population in tissue [66]. The development, maintenance, and contributions of adventitia, Mac^AIR^, and monocyte lineages to foam cell formation are outlined in Figure 2. Our work and others’ suggest that monocyte recruitment during these early stages dominates lesion progression and is required for plaque expansion [66,70,71]. In advanced lesions, others have shown in a series of elegant assays that proliferation of local monocyte-derived macrophages eventually becomes the primary source for expansion and maintenance of the foam cell pool in atherosclerotic plaques [72]. Data from advanced human samples investigating local plaque proliferation have also supported this possibility [73]. Thus, these data suggest that monocyte recruitment is likely a major driver of early lesion deposition and expansion, and this dependence transitions to favor macrophage proliferation in more advanced lesions. This shift might imply local metabolic rewiring according to substrate availability in the plaque microenvironment. Furthermore, a reduction in the proliferation of local plaque macrophages was a primary mechanism driving plaque regression in a mouse model, supporting the potential for substrate availability as a regulator of this function [74]. We propose that future investigations with new animal models allowing for fate mapping should be used to address this proposal, with a focus on plaque localization and regional contributions. 

Fate mapping approaches have sought to track monocyte entry and differentiation programs. A popular approach has been to use nondegradable bead uptake to track circulating monocyte entry into lesions at desired stages of disease progression or regression. Prior work found that monocyte recruitment occurs at all stages of disease progression [75,76]. While this may seem at odds with the observation that local proliferation controls foamy macrophage persistence in advanced plaque [72], these fate mapping data do not show the longevity or differentiation of monocytes in plaques at these different stages. To test the idea that monocytes are unable to support the foamy macrophage pool deep within advanced lesions, we performed a long-term monocyte fate mapping strategy during progressive or regressive conditions. By charting positions of monocyte entry at short time points and comparing them to the location of labeled cells after weeks of time within plaques, we were able to determine the ability of monocytes, as a population, to migrate within advanced lesions [77]. We observed that monocytes failed to penetrate deep within lesions, and that following initial recruitment, they were unable to perform substantial migration within plaque, regardless of progressive or regressive conditions. These data suggest that monocytes do not have substantial access to the deep regions in plaque where foam cells reside and support the notion that local proliferation maintains the foam cell pool, but that monocyte recruitment drives the superficial expansion of lesions from the plaque shoulder. However, these questions remain to be fully addressed and the application of new fate mapping animal models or live-imaging approaches may provide key supporting data to resolve some of the lingering issues regarding monocyte dynamics at early versus late lesions. In addition, new big data approaches, such as spatial sequencing, may help to elucidate differentiation and gene expression changes associated with residency in deeper layers of plaque. The factors that favor the switch from monocyte recruitment to macrophage proliferation in deeper plaque regions require further investigation.

### 4.2. Heterogeneity of Macrophages and Polarization in Atherosclerosis

Intravital imaging approaches have also been utilized to describe the migratory behavior of monocytes and macrophages within atherosclerotic lesions. A recent study using LysM^cre^ TdTomato^fl/fl^ reporter mice to label plaque monocytes and macrophages revealed that the majority of plaque-resident cells are sessile [77]. Motile cells were found in plaque shoulders, a region where one would expect monocyte entry. Because LysM promoter is active in monocytes, macrophages, neutrophils, and some DCs, the use of CCR2^gfp^ mice, in which monocytes are selectively labeled in the blood, confirmed that monocyte motility was detected specifically in plaque shoulders [77]. In an impressive follow-up study, investigators combined the use of CX3CR1^gfp^ and CD11c^yfp^ reporter mice to identify the coexistence of four different populations of macrophages in plaque [78]. Interestingly, the authors observed morphologic changes resembling extension and retraction of processes, termed dancing, associated with the expression of CX3CR1^gfp^, with or without the coexpression of CD11c^yfp^, but consistent with previous observations, they had nonmigratory behavior in plaque. Other cells expressing only CD11c^yfp^ with small, rounded morphology showed migratory behavior and may be consistent with monocyte-derived cells or, potentially, a DC subset [78]. The four cell types described display a differential transcriptomic signature associated with a unique cell shape and motility [78]. CX3CR1^+^ and CD11c^−^ cells highly expressed CD206 (Mrc1) and CD163, suggesting an alternatively activated phenotype of those macrophages. However, CX3CR1^−^and CD11c^+^ expressed many genes encoding for collagen and enzymes involved in extracellular matrix remodeling [78]. These observations are surprising because CD11c has been historically considered a proinflammatory macrophage marker, while collagen production is usually, but not strictly, attributed to anti-inflammatory macrophages. In addition, CD11c and CX3CR1 markers are not uniquely expressed by macrophages and monocytes but could potentially label a subset of DC, justifying the need for continued investigation. Overall, the dependence between these cell types for plaque development and differentiation programs are currently not well understood. 

Macrophage diversity within the homeostatic and diseased aorta has been of great interest to the field. The recent development of scRNA-Seq technology has allowed for the identification of cellular heterogeneity and rare cell populations isolated from tissue [79]. Three studies published in 2018 focused on immune cell diversity in steady state and at stages of disease progression [80,81,82]. These data describe multiple macrophage populations associated with a variety of inflammatory, anti-inflammatory, and remodeling gene programs, and further integration of these datasets has been performed to develop a meta-analysis of scRNA-Seq data providing greater resolution of clustering and rare cell identity [81,83]. By utilizing a fluorescent lipid labeling approach to separate foam cells from other macrophages in the plaque, Kim et al. described the gene expression profiles of cholesterol-loaded macrophages from atherosclerotic aorta, as well as nonfoamy macrophages from plaque. These data suggest that foamy macrophages fail to express highly inflammatory genes, but that these cytokines are expressed in the nonfoamy intima-associated macrophage pool [82]. These data are consistent with other studies suggesting that lipid-loaded macrophages fail to respond to inflammatory stimuli as efficiently as nonfoamy macrophages [84,85]. Beyond descriptions of foamy and nonfoamy macrophages from intimal lesions, these data also confirm the presence of Lyve1^+^ CD206^+^ macrophages associated with adventitia-originating cells and the absence of Lyve1^+^ cells from plaque [46,47,82,86]. Similar scRNA-Seq transcriptomic analysis has been performed in human plaque samples, describing the heterogeneity of total immune cells present in lesions and defining gene expression programs associated with human foamy macrophages [87]. Recent efforts from our group and others to compare mouse and human data to understand the shared heterogeneity of macrophage populations from plaque suggest considerable overlap in transcriptomic profiles between the foamy and inflammatory subsets in mice and humans [66,83].

Monocyte plaque entry generates a pool of plaque-resident macrophages that adopt multiple phenotypes according to their in situ localization and access to nutrients and cytokines. Metabolism and, in particular, glucose play a key role in monocyte generation. Indeed, increased glucose levels favor myelopoiesis [88], and pharmacological or genetic inhibition of glucose entry and metabolism prevents myeloid cell generation [55,89]. Through the use of selective genetic models (Lyz2^cre^ Glut1^fl/fl^), it was demonstrated that Glut1 meditates glucose entry into monocytes and macrophages. Glut1 myeloid cell ablation led to a massive decrease in glycolysis and pentose phosphate pathway (PPP) metabolites [54]. However, on an atherogenic Ldlr-deficient genetic background, Lyz2^cre^ Glut1^fl/fl^ mice had a similar plaque size and macrophage intraplaque content. The necrotic core area was increased, suggesting that monocyte/macrophage glucose metabolism is required for efficient death cell removal in plaque [54]. Glucose metabolism was also associated with effective macrophage polarization [90,91]. Alternatively (IL-4 stimulated) and classically (LPS and/or IFNγ) activated macrophages express selective markers. For instance, in mice, macrophages CD206, CD301, Relmα, Chi3l3, Lyve1, and Arg1 are increased upon IL-4 stimulation and associated with alternative polarization. On the contrary, CD11c and iNOS are induced in classically activated proinflammatory macrophages. Importantly, aspects of these polarization markers, including Arg1/iNOS, are not fully discriminatory in human macrophages. Comparisons between the induction of in vitro and in vivo macrophage activation programs have also emphasized potential difficulties in utilizing broad polarization descriptions of these populations [92]. Whether these polarization markers reflect on a specific activation state, local access to IL-4, or metabolites (such as glucose, fatty acids, or amino acids) remains to be fully established.

Whether a particular activation state dictates plaque macrophage and monocyte functions is an intriguing question. In a regression model, alternatively activated cells promoted plaque regression [93,94]. In this scenario, macrophage alternative polarization relied on Stat6. Indeed, Stat6-deficient macrophages failed to fully engage an alternative polarization and this was associated with compromised plaque regression [93]. Microscopy analysis identified a particular localization of alternatively activated macrophages in human plaque. These cells were detected near calcified areas [95], iron depots and hemorrhages [96,97,98], and highly vascularized zones [99]. These CD163^+^ macrophages participate in angiogenesis, and the use of CD163^−/−^ApoE^−/−^ mice suggests a proatherogenic role of those cells [99]. Of interest, proinflammatory macrophages are preferentially located on plaque shoulders, which are instable rupture-prone parts of the plaque [100]. The causal relationship between the presence of these cells and plaque stability remains to be established, and correlations between cells described in these studies and scRNA-Seq datasets have yet to be fully integrated. Continued use of spatial mapping approaches, at multiple stages of disease, will likely lead to important answers to these lingering questions.

### 4.3. Inflammatory Cytokine Production in Atherosclerotic Plaque

Historically, in advanced plaque, foam cells were considered highly proinflammatory and the contribution of monocytes and recent monocyte-derived macrophages to local inflammation was neglected. Using scRNA-Seq of total leukocytes from atherosclerotic aorta, we described the highest inflammatory cells to express CCR2, consistent with monocyte lineage cells [82]. Foam cells failed to be dramatic expressers of inflammatory cytokines [82]. These data suggest that monocytes may be key players in proinflammatory cytokine production, especially at plaque shoulder regions, beyond their roles in seeding macrophages within plaque. Further, inflammation has emerged as an attractive therapeutic target to prevent atherosclerosis development [101]. Primary proinflammatory cytokines produced by macrophages during atherosclerosis initiation and development are IL-1β, IL-6, TNFα, and CCL2. In the following section, we provide a brief discussion of their known roles in atherosclerosis, as deeper review articles have been recently published [102,103].

#### 4.3.1. IL-1β

IL-1β is a member of the IL-1 cytokine family containing a total of 11 members and 10 receptors. IL-1β is produced as a precursor cytokine lacking a signal peptide that requires cleavage in order to generate the bioactive form, which is exported in the interstitial space. In atherosclerosis, this process may be activated by the formation of intracellular cholesterol crystals capable of initiating the inflammasome complex [104]. IL-1β is produced by myeloid cells, including macrophages, monocytes, and neutrophils, as well as by arterial endothelial cells and smooth muscle cells [105,106,107]. IL-1β genetic ablation in mice, but not IL-1α, prevents plaque development [108]. The presence of IL-1β has also been detected in human plaques [109,110]. IL-1β promotes leukocyte adhesion on vascular endothelial cells [111,112]. Aortas obtained from atherogenic IL-1R1-deficient mice had decreased levels of the adhesion molecules ICAM-1 and VCAM-1 in comparison with IL-1R1-sufficient controls [113]. However, it was also reported that antibody-blocking experiments against IL-1β in animal models at late disease stages showed elevated plaque vulnerability by reducing smooth muscle cell remodeling and fibrous cap formation, thus challenging the potential of this pathway for treating patients with advanced disease [114]. The Canakinumab Anti-inflammatory Thrombosis Outcome Study (CANTOS) using IL-1β-targeting monoclonal antibody (canakinumab) demonstrated a lowered cardiovascular-event-related mortality rate in patients showing high plasma CRP levels [115]. However, and in agreement with the critical involvement of IL-1β during infectious diseases, canakinumab treatment was associated with increased susceptibility to infection. Thus, IL-1β blockade failed to increase the overall survival rate of the patients but acted as a proof of concept for the approach of targeting the immune system in addition to typical cholesterol management to reduce adverse cardiovascular disease outcomes.

#### 4.3.2. IL-6

IL-1β triggers many proinflammatory pathways, and among those, the IL-6 signaling pathway has attracted particular attention in the field of cardiovascular diseases. A genetic link between IL-6 plasma concentration and coronary heart disease has been established [116]. IL-6 signals through a membrane-bound receptor: IL-6R. IL-6R genetic variant was associated with decreased CRP levels and increased IL-6 concentration. In mice, exogenous IL-6 administration aggravated plaque lesions in comparison with untreated ApoE^−/−^ animals [117]. IL-6-deficient ApoE^−/−^ mice developed surprisingly larger plaques [118]. IL-6 deficiency was associated with decreased macrophage content and collagen staining. Pioneering work found that IL-6 mRNA expression was increased in atherosclerotic plaques [119]. IL-6 protein was colocalized in plaque with CD68 staining, suggesting that plaque macrophages produce this cytokine during atherosclerosis development [120,121]. Nevertheless, the role of plaque-derived IL-6 in disease progression remains to be fully established. 

#### 4.3.3. TNFα

Similar to IL-1β and IL-6, TNFα is found in cells inside the plaque [109]. TNFα-deficient ApoE^−/−^ mice had decreased plaque size compared with TNFα-sufficient ApoE^−/−^ animals, demonstrating the deleterious role of this cytokine during atherosclerosis development [122,123]. TNFα deficiency has been associated with decreased plaque inflammation, as illustrated by lower ICAM-1, VCAM-1, and MCP-1 mRNA expression in aortas [123]. This observation confirmed the previously demonstrated role of TNFα favoring lymphocyte adhesion to endothelial cells [124]. Bone marrow transplantation experiments demonstrated that immune cells are the main TNFα producers influencing plaque development [122]. In contrast, administration of anti-TNFα antibody increased plaque burden despite decreasing plasma proinflammatory cytokine (IL-6) and chemokine (MCP-1) levels [125]. Interestingly, TNFα neutralization induced lipid profile modulation with increased plasma triglyceride (TG) levels in comparison with control mice [125]. In a previous report, and despite no statistical difference detected, TNFα-deficient mice tended to have increased plasma cholesterol levels [122]. How precisely TNFα inhibition impacts TG levels, by regulating their production or metabolism, requires further investigation.

#### 4.3.4. CCL2 (MCP-1)

Lastly, the chemokine CCL2 is a primary mediator of monocyte recruitment, which drives the expansion of atherosclerotic plaques [18,126]. CCL2 is typically not expressed in nascent arteries but can be detected in plaque samples from human and mouse endothelial cells, smooth muscle cells, and myeloid cells [127]. Deficiency in CCL2 or CCR2 leads to dramatic attenuation of plaque progression [70,71,128]. Conversely, overexpression of CCL2 by the myeloid compartment was sufficient to exacerbate atherosclerosis progression [129]. Importantly, polymorphisms associated with the CCL2–CCR2 pathways have been linked to susceptibility to atherosclerosis and other cardiovascular diseases [130,131]. Therapeutic approaches in preclinical and clinical settings have shown effective blockade of the CCL2 axis as a therapeutic strategy [132,133]. Thus, targeting chemokine axes such as CCL2–CCR2, as well as many others that have been implicated in the pathogenesis of disease, is a leading candidate for treating atherosclerosis [103,133]. 

## 5. Conclusions and Perspectives

In conclusion, results from multiple studies support the role of monocytes and macrophages in promoting the progression and instability of atherosclerotic plaque. While dramatic advances have been made in understanding the heterogeneity and migratory potential of monocytes, many questions remain and will need to be addressed before targeted therapeutic approaches can be implemented. This includes a better understanding of the differentiation kinetics of monocytes within lesions and the contribution of monocytes at different stages of disease progression. Refined understanding of cellular interactions within atherosclerotic plaques, likely generated through multi-omics approaches to define cytokine and receptor pairs with cellular phenotypes as plaque progresses, will likely allow for this breakthrough. Current anti-inflammatory approaches are focused on the function of IL-1β, IL-6, and TNFα. However, the limited success of blocking IL-1β and the ubiquitous nature of these pathways for inflammatory responses may lead to disappointment. Expanding our understanding of nonoverlapping tissue-specific or disease-specific monocyte differentiation programs will allow for more targeted therapy, specific to cardiovascular disease, without inadvertently inhibiting broad inflammatory pathways. Critical questions regarding intraplaque metabolic programing and potential spatial constraints within plaque regions could be responsible for successfully developing approaches to modify the monocyte and macrophage response in plaque. Lastly, inclusion of additional translational studies is desperately needed to address whether observations made in preclinical mouse models replicate what occurs in human disease. Together, we believe that answers to the important questions highlighted in this manuscript will lead to the development of innovative approaches to combat and reduce the severity of atherosclerotic disease.
